# Boehringer launches first direct thrombin inhibitor: dabigatran (Pradaxa®)

**Published:** 2010-06

## Introduction

Dabigatran, an oral reversible direct thrombin inhibitor, already available in Europe and the United Kingdom, has been licensed for anti-coagulant use in orthopaedic hip and knee surgery in South Africa. At the launch of this novel anticoagulant, Prof Simon Frostick, professor of orthopaedics at Liverpool University pointed out that experience from clinical trials and in-practice experience has shown dabigatran to be a safe and effective agent in deep-vein thrombosis (DVT) prophylaxis.

Last year at the European Society of Cardiology (ESC) congress, dabigatran etexilate was introduced to cardiologists around the world in the RELY trial.^1^ This trial in 18 113 patients with atrial fibrillation showed that dabigatran at doses of 110 or 150 mg twice daily was as effective as warfarin in reducing stroke and systemic embolism. Major bleeding rates were lower than warfarin using the lower dose, and similar on the higher dose. The rate of haemorrhagic stroke was less with dabigatran than in patients receiving warfarin.

Public concern regarding ‘clot deaths’ in hospital have led to calls in the United Kingdom and South Africa for effective prophylaxis to be used in all at-risk patients. ‘Risk stratification protocols developed by the South African Society for Thrombosis and Haemostasis (SASTH) are now being used in all Mediclinic and Netcare private hospitals, and also in the state sector’, Prof Mervyn Mer, Department of Medicine at the Witwatersrand University noted. ‘In my view, a death from a venous thrombo-embolic (VTE) event following surgery or a hospital stay, where no appropriate thrombo-prophylaxis has been given, is entirely unacceptable’, Prof Mer declared.

Traditionally, new anticoagulant modalities are evaluated in the orthopaedic arena, as total knee or hip surgery carries a high risk of VTE and its fatal consequence, pulmonary embolism if anti-coagulation measures are not applied effectively and for a considerable time post surgery. ‘Risk of bleeding is always at the top of the list of orthopaedic concerns when evaluating new and existing anti-coagulants’, Prof Frostick noted.

The prevalence of VTE is difficult to pinpoint as it is often asymptomatic, undiagnosed and unrecognised at death and post mortems are infrequently done. Using an epidemiological model, one study^1^ estimated the number of VTE deaths in major countries in Europe to be in the order of 370 000. Almost threequarters of these deaths were hospital acquired; very few actually occurred in the orthopaedic arena, as in this discipline, the focused use of anti-coagulants has reduced the number of VTE deaths.

In the United States, a study^2^ on the characteristics of patients operated on between 1990 and 2004 for hip or knee orthroplasty showed an in-hospital mortality rate of 0.35%. Pulmonary embolism was the most frequent adverse event.

Middle-aged women undergoing knee or hip replacement are particularly vulnerable and a United Kingdom study has found that one in 45 women admitted between 1996 and 2001 for surgery was re-admitted to hospital with venous thrombo-embolism during the 12 weeks after surgery.^3^

While in the 1990s it was possible to argue that prophylaxis with anti-coagulants (LMWH, unfractionated heparin or warfarin) did not reduce symptomatic thrombo-embolic events,^4^ the advent of new agents has led to a body of evidence that VTEs are preventable and many guidelines are now available for the use of agents in both medical and surgical patients.^5^

Racial variations exist: a recent review has noted African-American patients have a significantly higher rate of incident VTE leading to pulmonary embolism.^6^ There are no prevalence data on DVT in African patients, but it is clear that VTE is not solely a caucasian phenomenon.

‘Aspirin is ineffective in VTE prophylaxis and with new designer-molecule anti-coagulants such as dabigatran, which target specific events in the coagulation cascade, we do not need to use the older sledge hammer-like anticoagulants’, Prof Frostick pointed out. ‘When selecting an anti-coagulant, bleeding rates are a major factor; but tolerability, efficacy, simplicity and flexibility are valuable features of the newer anti-coagulants’, he stressed.

‘Dabigatran is effective in preventing VTE events, with similar bleeding rates to enoxaparin in both hip and knee surgery.^7^,^8^ Its time-to-peak levels is six hours following surgery, due to surgeryinduced delay, which allows for clotting around the wound site to be more effective. Normally a half dose is given initially, either 75/110 mg on the evening of surgery’, Prof Frostick pointed out.

The trials with dabigatran were designed as non-inferiority trials with preset margins. In the RE-MODEL trial, dabigatran 150/220 mg was compared to subcutaneous enoxaparin 40 mg once daily for six to 10 days, starting on the evening before surgery. Patients were followed up for three months. Both doses of dabigatran were non-inferior to enoxaparin on the basis of the pre-specified non-inferiority criteria. The incidence of major bleeding did not differ significantly between the three groups (dabigatran 150, 200 mg and enoxaparin 40 mg). There were no significant differences in the incidence of liver enzyme elevation or acute coronary events.

‘It should be noted that in high-risk patients undergoing total knee replacement, a reasonable approach would be to continue prophylaxis for 35 days; a similar period to that recommended for hip replacement by the American College of Chest Physicians (ACCP) guidelines and the UK NICE guidelines 2010’, Prof Frostick added.

‘When comparing rivaroxaban and dabigatran, independent analyses suggest a superior action of rivaroxaban to enoxaparin at the cost of an increased bleeding risk. The rivaroxaban data on major bleeding events is not truly representative of the actual bleeding rates as this category of major bleeds did not include site bleeding data unless it required re-operation’, Dr Frostick pointed out. ‘In the trials of dabigatran, site bleeding was included in the definition of major bleeds. With dabigatran in both knee and hip surgery, most of the major bleeds occurred at the surgical site; for example, in RENOVATE, 91% of the major bleeds were at the surgical site while some 46% (150-mg dose) and 56% (220-mg dose) of major bleeds occurred before drug administration.’

In the published literature, direct comparisons of rivaroxaban and dabigatran are not possible. However, in interpreting anti-coagulant data and comparing these drugs to enoxaparin as far as safety is concerned, it is clear that rivaroxaban with its superior efficacy carries a consistently higher bleeding risk. This view is supported by independent evaluations undertaken by the FDA and those done for the UK standard, NICE. ‘The NICE technology guideline in fact comments that with regard to efficacy, the two agents are similar, but there is a tendency to increased bleeding with rivaroxaban’, Prof Frostick said.

The availability of two doses of dabigatran is useful when dealing with more fragile patients such as the elderly (> 75 years) and those with moderate renal insufficiency. ‘In the period of real-life clinical usage of these new entities, we need to do our own post-marketing surveys and we should not be complacent about any new clinical agents’, Prof Frostick concluded.

The panel of South African experts added to the presentation with their experience of using dabigatran in the clinical trials locally, which increased patient numbers significantly in both the RE-MODEL and RE-NOVATE trials.

‘Mobility in the older patient undergoing surgery is very important and we should understand the need for anti-coagulation in these patients for an extended period of time’, Prof Barry Jacobson, head of haematology at the Witwatersrand University pointed out. ‘The clinician should watch out for non-steroidal antiinflammatory usage, and protease inhibitors for HIV treatment can also complicate the expected anti-coagulant action of dabigatran’, he warned.

With regard to reversing the effects of dabigatran when required, activated charcoal and recombinant factor VIIA areuseful, while in an emergency situation, the patient can be dialysed. The advantages of an oral medication such as dabigatran cannot be ignored as patients are now sent home very early after surgery, normally within three to five days.

## References

[1] Cohen AT (2009). Thromb Haemost.

[2] Memtsoudis SG (2010). J Arthroplasty.

[3] Sweetland S (2009). Br Med J.

[4] Warwick DJ, Whithouse S (1997). J Bone Joint Surg.

[5] Geerts WH (2008). J Bone Joint Surg.

[6] White RH (2009). Thromb Res.

[7] Eriksson BI, Dahl OE, Rosencher N, Kurth AA, van Dijk CN, Frostick SP (2007). J Thromb Haemost.

[8] Eriksson BI, Dahl OE, Rosencher N, Kurth AA, van Dijk CN, Frostick SP (2007). Lancet.

